# Western Medical Schools

**Published:** 1836

**Authors:** 


### Art. VI. Western Medical Schools.

*A Report made to the Legislature of Kentucky on the Medical Department of Transylvania University*, Feb. 15, 1836. By Charles Caldwell, M. D.

*A Catalogue and Circular of the Officers, Professors and Students of the Willoughby University of Lake Erie*, 1836.

*Introductory Lecture on the importance of the information derived from Medical Science, in improving the condition of a Country, &c.* By Ed. H. Barton, M. D. Professor of the Institutes and Practice, and of Clinical Medicine, in the Medical College of Louisiana, &c., &c., New Orleans, Dec. 1835.

*An introductory Lecture, in which is considered the political importance of Medical Schools.* By W. Byrd Powell, M. D., professor of Chemistry and Pharmacy, in the Medical College of Louisiana, &c. New Orleans, 1835.

In the pamphlets, the titles of which we have just enumerated, we find much that should gratify the profession in the West. The first affords evidence, that the oldest institution of the Great Valley, maintains herself on the elevation to which she rose, with a rapidity, almost unequalled, while the latter indicates a tendency in the public mind, to establish institutions for medical education, in regions where they are needed, and where but a few years since, such enterprizes would have been thought altogether chimerical,—we refer to the shores of Lake Erie and the Gulf of Mexico. We view these efforts with the greatest approbation, inasmuch, as they evince, in the profession and society at large, great activity of feeling, and must, by reciprocally stimulating each other to intense effort, produce results the most beneficial to science and humanity.

The character and genius of free governments, confide to the great principle of unrestrained and encouraged emulation, the whole subject of elementary and professional education. The most important business of the world, after the cultivation of the soil, is the cultivation of the mind and heart—the most useful class in society, are the agriculturalists, the most influential, the teachers. As the number of the former increase, the price of sustenance is diminished, consumption advanced, and human enjoyment promoted; as the latter multiply, the price of tuition diminishes, the professors make intenser exertions, learning becomes more fashionable, ignorance more contemptible, and the number of the educated in all the departments of society, proportionably greater.

In the sparsely settled portions of our new country, where school houses are few in number, many children grow up in ignorance, who would be educated, if they were reared within sight of one of these sylvan temples of primary learning. In like manner, when students of medicine are near to medical schools, they avail themselves of advantages, which but few of them seek, at the expense of a journey of several hundred miles. Before the University of Pennsylvania was organized, but a small number of our physicians received a collegiate medical education, because to that end, a voyage across the Atlantic was required; and previous to the establishment of Transylvania University, thirty years afterwards, an inconsiderable part only, of those who commenced the practice of medicine in the West, graduated.

There was a time, when superficial thinkers regarded the former of these seminaries, as sufficient for the nation! But although it grew, with a sound and regular growth, to a magnitude which made it an honor to the new world, other institutions, in part from the power of its example, came into existence; and from Maine to Carolina, the sea board was dignified with medical schools, in which hundreds were annually taught, who would not have visited our *alma mater*. In like manner, while Transylvania has been advancing to an elevation, that has conferred distinction on the West, other establishments have started into existence, and she now finds herself in the centre of a system—the most brilliant luminary of a new constellation.

Passing by for the present, those members of this group, which are found in *this* portion of the western firmanent, we may refer to the new stars that are rising, as it were from the waters of the Gulf and the Lakes, and hail them as auspicious to the public good. The diseases which prevail in the basins of the great Mediterranean seas, relatively high up in the north, have their peculiarities, and the physicians who are to treat them, should, if possible, be instructed by teachers, who have experience of what they are to inculcate. The population of the shores of the Lakes, is increasing with a rapidity almost unequalled, and in a few years will require as many physicians as could be educated in a single school. Western New York, North Western Pennsylvania, Northern Ohio, Indiana and Illinois, the whole of Michigan and Wisconsin, and the South West of Canada, ought at this time, indeed to furnish an adequate number of medical students, to support a respectable school on the Lake shore, and such an one, we doubt not, will be speedily established. Whether that commenced at Willoughby two or three years since, the circular of which is now before us, will become such a school, we are not called upon to predict, but we know no reason why it should not. It has five professors, the text books which they recommend are, on the main, well chosen; the catalogue before us presents the name and residences of 23 pupils, and a list of 5 graduates for the session of 1835–6, and the president and secretary of the Board of Trustees, with whom, only, of all concerned, we have the pleasure of an acquaintance, are gentlemen of respectability and enterprize; *prima facia*, we see no obstacle to the success of the undertaking, and as far as the humble influence of this Journal can, it will be found to promote that object.

The Medical College of Louisiana, in New Orleans, was chartered in the winter of 1834–5. Its class was of course small, but the session which is just over, was attended, as we learn, by 22. This number, as well as that in the Willoughby school just quoted, may seem small, but let it be remembered that the schools of Philadelphia and Lexington, opened in a manner equally humble. We cannot conceive why a respectable Medical College should not be established in N. Orleans. But a few years since, the project of instituting a medical school in Charleston, was regarded as chimerical—now she has two, both of which, we believe, are in a flourishing condition.

The number of physicians required in the maritime parts of Louisiana, Mississippi and Alabama, to say nothing of West Florida and Texas, the latter of which must be supplied from the United States, is great, and physicians educated in the

Delta of the Mississippi, would, *cæteris paribus*, be much superior to those instructed by teachers inexperienced in the diseases peculiar to that region. The great commercial emporium of several millions of people — New Orleans — must forever afford an amount of clinical practice, and a supply of dissecting room subjects, unequalled perhaps by any other city in the union. It is true, that from the mildness of the winter, the latter cannot be long preserved; but this evil will be effectually counteracted by their number.

On this subject prof. Powell holds the following language, which we quote as a specimen of his style and thinking, and a correct statement of the argument in favor of adapting schools, to all the great localities of a country.

"Each district of country, from a difference of latitude, topography and other local contingencies, is, sometimes, visited by diseases peculiar to it, and always by particular forms or types of those which are common to many or all.

"For these peculiarities to be understood, they must be observed in the very spot where they occur. Consequently, the student should have advantages of instruction from those who are in the constant habit of observing them, reflecting on their causes and character and administering to the relief of those who are afflicted with them. In illustration of this doctrine, every one will admit the palpable absurdity of sending a student from Egypt to London to acquire a knowledge of the plague—or from Boston to this city to study the flattering and discouraging mutations of consumption—or from this city to Boston to learn the nature and treatment of Yellow Fever, or from our Charity Hospital to our interior Medical Schools to study clinical Medicine and Surgery; there being in those places, comparatively, very few opportunities for either.

"The principles of medicine being, universally the same, it may be contended that he, who understands them, is qualified to practice in any place; but it must be recollected that we do not, with the acquisition of principles, obtain a knowledge of all their special applications. Add to this fact, the difficulties which contingent circumstances produce, by acting upon the innumerable susceptibilities of our nature, and it becomes evident that the application of medical principles to the treatment of the diseases of any given district, requires a special study.

"Now the question is, shall this *necessary study* be made during the period of ordinary pupilage, and under the direction of competent teachers, or after the commencement of professional practice, and that too, under the responsible considerations imposed upon the practitioner by the danger of injuring his professional reputation, through unsuccessful practice, with the consequent loss of many valuable members of society."

The professor goes into a comprehensive survey of the advantages to society, of the cultivation of Medical knowledge, especially of Chemistry, which it is his own proper business to teach, but the plan of this article does not permit us to follow him.

The object of professor Barton, is to show the importance of cultivating medical science, with a view to its application to public hygeine ; and of consequence the value to the people of Louisiana, of cherishing a medical school. In reference to the latter, we make the following excerpt :

"With a peculiar climate and physical condition—with diseases and wants incident to this peculiarity—with a happy location which nature has pronounced past all rivalry—the commercial mart of an illimitable region, which the perfection of navigation, the wonders of steam, the facilities of rail roads in surmounting the obstacles of distance, all supply branches of that great circulatory system, whose heart is *here*, we find ourselves in a position, where it is indispensable we should rely upon ourselves. Here we are called upon, apart from our love of independence, to rely upon our own resources; resources, too, growing out of our peculiar wants, and which can be known only to ourselves.

"To become acquainted with this condition, to alter it where productive of disease, to point out those which are peculiarly producive to health and prolongation of life—to adapt man in living, clothing, exposure and circumstances, to the position in which Providence has placed him—is the peculiar province of our profession."

The necessity of science, enterprise and industry, to the preservation of the health of communities, is set forth by the professor, in the following remarks :

"It should be as gratifying to the pride, as it is flattering to the industry and intellect of man, that through their constant efforts only, the salubrity of any spot (not salubrious from position) is maintained ; when these are relaxed, or when prosperity and civilization. decline, the seeds of disease, are, as it were, immediately deposited in the earth. There is scarcely a nation of any note mentioned in history, whose progress and decline are not illustrative of this truth. In the flourishing condition of empires, disease has been kept at bay—industry and cultivation have kept pace with population—the arts and sciences have flourished, and man has fulfilled the great end of his being. With the decay of the arts and the innervation of a people, cultivation has been abandoned—negligence has supplied the place of industry, and the mouldering columns and dilapidated palaces are the sure fore-runners of the pestilence that sweeps its desolating besom over the land, and finishes that which man has commenced. The sombre aspect of the Ottoman empire and the flourishing condition of Great Britain, furnish impressive pictures of the truth of these remarks, the former being in the most neglected and sickly state, the latter the best cultivated and most healthy country of Europe. It is thus that fate itself, fore-domed by negligence and ignorance of invariable physical and moral laws, advances to destroy the cherished pride of many ages. Rome, once the queen of cities, is following the fate of Babylon, and from the same cause is daily diminishing in population. Pestilence advances from street to street, and has already become the sole tenant of some of its finest palaces, temples and churches. Rome, indeed, might be singled out, as affording in itself, and as a warning to us, a history of most that is interesting in the police of health. When still the capital of the world, in spite of pestilence, she overflowed with population, and the disadvantages of her position were counteracted by the activity and moral excitement of her inhabitants, the drainage of marshes, the width and durability of her paved streets, and the abundant supply of pure water, from her numerous aqueducts, for baths and other domestic purposes."

The author proceeds to make an application of his principles to the Delta of the Mississippi, and especially, to the site of New Orleans:

"And what does not our own country owe to the advantages of draining, clearing and the influences of agriculture? By it spots that were untenantable or fatal to the early settlers, afford healthful homes to thousands—the primeval forest has given way before the axe of the husbandman—the sickness of the early settlements, with their half cleared and half cultivated fields, have disappeared before a thorough and perfect reclamation and cultivation. The same effects would follow with many additional benefits, the clearing of our own swamps, by removing the sources of moisture and evaporation—our heavy dangerous dews, and those obstructions to ventilation, without which there can be no health any where; general salubrity would diffuse its blessings upon us, population would increase, and all the comforts and enjoyments of a highly advanced and progressive condition, would be poured upon us. Cast your eyes for a moment, upon what you have already done; the very ground on which you now tread, was but a few years since a great unsightly morass—the home of the alligator, the retreat of the bear and tiger, where the contest was, whether the watery element or the evergreen cypress with its funereal foliage, should hold dominion over the country. Now view the contrast! a city springing as by enchantment from the subdued marsh, with scarcely a rival in the magnificence of its buildings—the extent of its improvements and resources—the lofty enterprise and intelligent foresight of its inhabitants, stretching her arms of conquest—of cultivation—of commerce, over regions on which the sun never sets, and I wish I could add, as I hope soon to do—equally remarkable for the extent and usefulness of her literary endowments. But yesterday, it was in the cradle of its existence—the outline of its future is lost in a splendor unsurpassed, which the racked eye and bewildered fancy in vain exerts itself to realize or circumscribe. But mark, the splendid outline of this prophetic future, is only to be filled up through industry—through toil—by the fulfillment of the moral as well as physical laws. Indolence then is forbidden by the decrees of Providence, and this is legibly written in the character of your country. Your reward shall be commensurate with your exertions—*nil sine magon vita labore dedit mortalibus*, noble is the incentive, rich the return, and without it, disease, degradation, poverty, slavery."

Leaving the new school of the South to the fostering care of that industry which is here so earnestly recommended, and which cannot fail to raise it into respectability and usefulness, we turn to our western *alma mater*, and find ourselves forcibly carried back to the days of her first *lactation*. THE MEDICAL DEPARTMENT OF TRANSYLVANIA UNIVERSITY, was organized in the autumn of 1817. Its first Faculty consisted of five professors, Dudley, Blythe, Richardson, Overton and Drake. Its class numbered 20, and at the end of the session, just 18 years ago, the degree of Doctor of Medicine was conferred on one candidate, Mr. McCullough, a native of Lexington. This was the first Medical Commencement ever held west of the mountains. During the present spring, the same degree has been extended by five different institutions, to 130, and the number of students in attendance was 500—facts which speak well for the advancement of medical science in the Western wilderness.

The medical lectures in Transylvania, were suspended in the year 1818, and resumed in 1819;—the reorganized faculty being composed of Drs. Dudley, Richardson, Blythe, Brown and Caldwell—the number of pupils, 38. From this time, its development was most rapid, to the autumn of 1825, when it numbered 281, since which, it has fluctuated between 152 and 262. For the last three years, it has varied but two from 260. The aggregate number of students from its foundation, has been 3350, one half of which, we may fairly presume, would have gone into practice without a University education, if this school had not been established. In 1823, Dr. Drake was reappointed into this institution; in 1825 Dr.

Short, in 1827 Dr. Cooke, and 1830 or 1831, Dr. Yandell. The Faculty now consists of Drs. Dudley, Caldwell, Richardson, Short, Cooke, Yandell and assistant professor Peter. It is worthy of remark, as it is creditable to those who have, at various times, constituted the Medical Faculty of Transylvania, that its distinguished success has been independent of any other extraneous aid, than $5000, from the State of Kentucky, and an equal sum from the citizens of Lexington.

The object of Professor Caldwell's report or rather address, to the Legislature of that State, was to obtain an appropriation of $10,000 for the purchase of additional *materiel* for instruction. His application was supported by a compact and powerful argument, which we think ought to have been irresistable ; but that honorable body we believe, thought otherwise, and contented itself with giving to the learned and zealous professor, a respectful audience. We shall extract from it, as sustaining our views on the necessity and propriety of various schools in various localities, the following proposition which the professor informs us he presented to the General Assembly of Kentucky, 15 years before, with his late commentary upon it.

"*The School being established in the great Western Valley, will throw much new and important light on the diseases of that Valley, which must lead to a more rational and successful treatment of them.*

" This view, which was but *prophesy* when presented, and which few received with entire credit or favor, is *history now*. In evidence of its correctness, the pupils of Transylvania, are, as a very general rule, the ablest and most successful practitioners of their age, both in medicine and surgery, in the Mississippi Valley. However unacceptable and perhaps displeasing to some this assertion may prove, experience and observation testify to its truth. Though I make it therefore fearlessly ; yet, in doing so, I mean neither offence, condemnation, nor disparagement toward any physician, or any school. My object is to state what I know to be true. The statement, moreover, is but a *tribute of justice* to the sons of Transylvania, for their devotedness to their profession, the high standing they maintain in it, the benefits they confer on their fellow men in the practice of it, and the credit they reflect on their Alma Mater. *And I glory in awarding it*. Having first by persevering industry as pupils, rendered themselves competent to their professional duties, they are now faithfully performing them as practitioners. A knowledge of this induces their fellow citizens to confide in them and employ them. Hence the ascendency they so usually attain. Another leading cause of this result is plain. The professors of Transylvania being familiar with the nature and treatment of western complaints, can best communicate a knowledge of them to their pupils. For this reason alone, the School is worthy of every form and degree of patronage the public can bestow.

"Apart moreover from all other considerations, the period has arrived, and seems to be mature, when the western people should patronize and encourage western institutions alone. In no other way can such institutions be brought to the perfection they are destined to attain. Nor can they become otherwise productive of the full amount of usefulness, for which they are intended, and to which they can be made competent. To say nothing then of western pride, which should prompt the desire to become less dependent on eastern resources, the measure is called for by the INTEREST OF THE WEST. The Valley of the Mississippi is believed to contain between four and five millions of inhabitants, and is increasing in wealth, population, and power, with a rapidity undreampt of in any other section of the Union. It is but manly in it then to throw off its minority, proclaim its literary and scientific independence, and set up for itself. And I confidently trust that the day is near at hand, when the proclamation will be issued, and carried into effect. The enterprise is easy ; and, I repeat it, that the GREAT WESTERN COMMUNITY owes it alike to its INTEREST AND ITS HONOR. Within less than half a century, the preponderance of power in the Union will be in the Mississippi Valley. Nature herself, by the unrivalled magnificence of her western works, proclaims the "coming event," which already begins to "throw its shadow before." Let the people of the valley then, by providing for every form of mental cultivation, prepare themselves for the wise and virtuous exercise of this power, when in time it shall become theirs ! In this way alone can they discharge their duty to themselves and their country, their contemporaries and posterity !

Cherishing as we do a sentiment of respect for Transylvania, the pioneer institution of the West, we concur in almost every thing which the learned professor has advanced in her favor; but successful as her efforts in the cause of medical education have been, we do not look upon the organization and labors of her Medical Department, as faultless, nor is it precisely according to the fact, to affirm, as professor Caldwell, does (p. 32,) that the "*institution has acquitted itself to* ENTIRE *satisfaction for sixteen years, and done more, much more, on five thousand dollars, than any other similar school in the union, has done on seven-fold the sum.*" The Jefferson Medical College at Philadelphia, established under the walls of the University of Pennsylvania, when it had more than 400 pupils, without, as we believe, having received a dollar from the state, attained, during the past session to 370 pupils, an equal or greater number than the University, and nearly 100 more than Transylvania. The number of her graduates, the present year, was 14 or 15 greater than that of her elder sister, making her, indeed, the first school in the continent, although she came into existence, under circumstances the most adverse, six years after the present organization of the Transylvania Faculty. We make this statement in no spirit of detraction, but of justice; and to evince, that although western in our feelings, we are not insensible to that which is meritorious in the east.

In conclusion, we feel it a duty, to say something on the policy which should govern our schools. Increase in their number, will generate competition for pupils, which may either lead to an elevation or a depression of the standard of excellence. When rival institutions seek to surpass each other, in the value of their instruction, and to acquire superiority of numbers by deserving it, the rivalship is a public blessing; but if, on the other hand, they relax in their requisitions, and place their honors on a lower level, they soon become a curse to the community. Unquestionably, it is *lawful* — we use the word without a reference to statutory enactments—for any association of physicians to get up a medical school, but it is *not* lawful — that is, not compatible with the welfare of society, nor the dignity of the profession — for them to seek patronage by a system of underbidding, or, what is still worse, easy examinations. When a new school is started, it should recommend itself by some improvement on existing systems, so as to hold out, with justice, the promise of making better physicians, than the old; while they should protect themselves against increased competition, by rising in the requisites to graduation, as often as an additional competitor enters the list — thus maintaining their relative superiority of numbers, by actual and increasing superiority of instruction. When all parties act on these principles, they operate under a sentiment of emulation; and every new college becomes a new stimulus to those already in existence, while they in turn react on it, and destroy all hope of success, except it puts forth upon the object, extraordinary powers. In such a contest, weak men can do nothing, and this is the test of the truth of what we advance; for any system which banishes, the infirm of intellect, purpose, or perseverance, from our halls of learning, is good.—How far this principle has governed the organization and exercises of the medical schools of the West, and, indeed, of the Union, we shall not undertake to inquire; but it would be as rash, to affirm, that the best talent of the American profession is to be found in our seminaries, as that their organization does not imperiously call for revision.

